# Forecasting seizure likelihood from cycles of self-reported events and heart rate: a prospective pilot study

**DOI:** 10.1016/j.ebiom.2023.104656

**Published:** 2023-06-16

**Authors:** Wenjuan Xiong, Rachel E. Stirling, Daniel E. Payne, Ewan S. Nurse, Tatiana Kameneva, Mark J. Cook, Pedro F. Viana, Mark P. Richardson, Benjamin H. Brinkmann, Dean R. Freestone, Philippa J. Karoly

**Affiliations:** a School of Science, Computing and Engineering Technologies, Swinburne University of Technology, Melbourne, Australia; b Department of Biomedical Engineering, The University of Melbourne, Melbourne, Australia; c Seer Medical, Melbourne, Australia; d Department of Medicine, St Vincent's Hospital Melbourne, The University of Melbourne, Melbourne, Australia; e Graeme Clark Institute, The University of Melbourne, Melbourne, Australia; f Institute of Psychiatry, Psychology and Neuroscience, King's College London, London, UK; g Centre for Epilepsy, King's College Hospital NHS Foundation Trust, London, UK; h Centro de Estudos Egas Moniz, Faculty of Medicine, University of Lisbon, Lisbon, Portugal; i NIHR Biomedical Research Centre at South London and Maudsley NHS Foundation Trust, London, UK; j Bioelectronics Neurophysiology and Engineering Lab, Mayo Clinic, Rochester, MN, USA

**Keywords:** Epilepsy, Seizure forecasting, Seizure cycles, Multidien cycles, Wearable, Heart rate

## Abstract

**Background:**

Seizure risk forecasting could reduce injuries and even deaths in people with epilepsy. There is great interest in using non-invasive wearable devices to generate forecasts of seizure risk. Forecasts based on cycles of epileptic activity, seizure times or heart rate have provided promising forecasting results. This study validates a forecasting method using multimodal cycles recorded from wearable devices.

**Method:**

Seizure and heart rate cycles were extracted from 13 participants. The mean period of heart rate data from a smartwatch was 562 days, with a mean of 125 self-reported seizures from a smartphone app. The relationship between seizure onset time and phases of seizure and heart rate cycles was investigated. An additive regression model was used to project heart rate cycles. The results of forecasts using seizure cycles, heart rate cycles, and a combination of both were compared. Forecasting performance was evaluated in 6 of 13 participants in a prospective setting, using long-term data collected after algorithms were developed.

**Findings:**

The results showed that the best forecasts achieved a mean area under the receiver-operating characteristic curve (AUC) of 0.73 for 9/13 participants showing performance above chance during retrospective validation. Subject-specific forecasts evaluated with prospective data showed a mean AUC of 0.77 with 4/6 participants showing performance above chance.

**Interpretation:**

The results of this study demonstrate that cycles detected from multimodal data can be combined within a single, scalable seizure risk forecasting algorithm to provide robust performance. The presented forecasting method enabled seizure risk to be estimated for an arbitrary future period and could be generalised across a range of data types. In contrast to earlier work, the current study evaluated forecasts prospectively, in subjects blinded to their seizure risk outputs, representing a critical step towards clinical applications.

**Funding:**

This study was funded by an Australian Government National Health & Medical Research Council and BioMedTech Horizons grant. The study also received support from the 10.13039/100001605Epilepsy Foundation of America's ‘My Seizure Gauge’ grant.

**Research in context**

**Evidence before this study**

Before conducting this study, we searched the literature for any studies that investigated non-invasive seizure forecasting in people with epilepsy. We used Google Scholar and Web of Science with search strategies that combined one or more of the terms “epilepsy”, “seizures”, “forecast”, “predict”, “non-invasive”, “wearable” and “prospective” with no restrictions on language or dates. Across more than 6000 publications, only three groups conducted prospective evaluation of any type of seizure forecast (invasive or non-invasive). One study used an invasive intracranial implant device (NeuroVista) to predict seizures in a prospective trial of 15 patients. Another study conducted a prospective evaluation of self-prediction (where individuals self-rated their likelihood of having a seizure the next day from 0 to 100) using an electronic seizure diary app but did not evaluate individual seizure risk in real time. One other study prospectively validated the utility of a mobile seizure diary to determine whether a patient's condition in a given month was improved, worsened or unchanged compared to the previous month, with application as a clinical support tool. Numerous studies investigated seizure forecasting using retrospective electroencephalography (EEG) recordings but used either long-term invasive EEG or short-term scalp EEG and so were not considered as wearable forecasting systems. A number of studies evaluated the long-term performance of individual seizure risk forecasts using retrospective data recorded from non-invasive, mobile or wearable devices, primarily mobile diaries, smartwatch or armband devices. This volume of evidence based on retrospective data demonstrates the potential of wearable forecasting and highlights the value of a study to prospectively evaluate seizure risk forecasting.

**Added value of this study**

This study validated a mobile and wearable seizure risk forecasting app in a pilot study of 13 participants (adults with diagnosed epilepsy). The seizure risk forecasting algorithm is freely and publicly available in a mobile app (Seer App, Seer Medical Pty Ltd, Australia). Subject-specific risk forecasts were developed using multiday cycles of seizure times and heart rate. Performance was evaluated using self-reported seizures (mean: 125) over long-term recordings (mean: 562 days). Notably, forecasts were evaluated in a prospective, blinded setting for 6 of the 13 participants (the remaining 7 were lost to follow up). During validation, the best forecasts achieved a mean area under the receiver-operating characteristic curve (AUC) of 0.73 with 9/13 participants showing performance above chance. Prospective evaluation of forecasts showed a mean AUC of 0.77 with 4/6 participants showing performance above chance.

**Implications of all the available evidence**

This study prospectively evaluated mobile and wearable seizure risk forecasts in a cohort of 6 participants (who were blinded to forecast output), highlighting the prospective utility of a non-invasive device to forecast seizure risk. Results demonstrated forecasts were reliable for most individuals, with comparable performance to existing studies, and robust over time. The existing literature, in combination with this prospective pilot study, demonstrates that non-invasive seizure forecasting is now ready for clinical translation. Future work will initiate clinical trials of a mobile and wearable seizure risk app to improve patient outcomes, schedule epilepsy monitoring, or therapy titration.

## Introduction

Seizure forecasting is highly desirable for people living with epilepsy^1^ as it could enable the modification of daily activities and access to early interventions (such as fast-acting medication or neuromodulation) to prevent injuries and deaths.^2^ Early studies demonstrated the utility of electroencephalography (EEG) to detect and predict seizures.^3^, ^4^, ^5^, ^6^ However, some forecasting results showed poor generalizability due to a lack of long-term data and enough seizures.^7^ A first in human trial provided patient-specific seizure prediction algorithms and showed the possibility of seizure prediction using long-term continuous intracranial EEG data.^5^ Following this landmark trial, advances have been made in seizure forecasting using multi-day rhythms of epileptic activity.^8^, ^9^, ^10^, ^11^, ^12^, ^13^

Cycle-based models can generate promising forecasting results.^9^ Several recent studies provided personalised forecasts using cycles of seizure occurrence,^11^^,^^14^ interictal epileptiform activity (IEA),^8^ and EEG biomarkers of cortical critical slowing.^12^ Unlike cycle-based models, black-box machine learning models predict seizure likelihoods by fitting parameters using available datasets but have not yet been able to project the estimate of seizure likelihoods into future weeks or months. Additionally, cycle-based models may help avoid over-fitting by using a small number of parameters.^14^ Self-reported seizures are usually noisy and inaccurate as patients may miss events or report events that are not seizures.^15^ Machine learning models based on self-reported events alone could fail to capture true seizure likelihoods due to overfitting to patients’ reporting errors. On the other hand, since self-reported events generally align to the same underlying cycles as electrographic events,^16^ cycle-based models may be more robust to noise or reporting bias.

Forecasting algorithms using long-term EEG data may not be appropriate for some users since invasive devices may have unacceptable risks or costs.^1^^,^^17^ Although sub-scalp EEG devices are useful for seizure forecasting,^18^, ^19^, ^20^ even minimally invasive implanted devices are not desirable for many users.^1^ Currently, there is great interest in using non-invasive devices to monitor biomarkers of seizure risk. Several commercial non-invasive devices are promising in seizure detection^20^ and forecasting.^17^^,^^21^ Non-invasive wearable devices including detecting blood volume pulse, body temperature, cerebral oxygen saturation, and heart rate (HR) can be applied to predict seizures.^2^^,^^22^, ^23^, ^24^ More recent studies have also related periodic wearable physiological signals, such as HR, electrodermal activity, and temperature to seizure risk cycles.^25^^,^^26^

It is well established that cycles of seizure occurrence and IEA are related to seizure risk.^8^^,^^11^, ^12^, ^13^^,^^27^ Additionally, underlying biological cycles may also be independent predictors of seizure occurrences. Karoly et al. suggested that HR cycles were found in 76% of 26 participants, with 100% circadian, 88% weekly, and 92% monthly cycles.^25^ More recently Gregg et al. also found similar cycles of HR and multimodal wearable signals were related to cycles of electrographic seizure risk.^26^ A seizure forecasting algorithm using multiple HR cycles was proposed by Stirling et al., and the results showed that seizures could be predicted above chance in 11 participants.^24^

This study presents a forecasting method using multi-day cycles recorded from wearable devices, which is generalizable across different signal modalities. The power of this approach is that it enables existing methods of cycle detection from multimodal data to be combined within a single, scalable algorithm. This approach differentiates the current study from a previous wearable forecasting research,^2^^,^^24^ which has used more complex, black-box machine learning models. Here we validate forecasting performance using longitudinal data from seizure mobile diaries and a smartwatch. Critically, forecasting performance was also evaluated in a blinded, prospective setting, using long-term data collected after algorithms were developed.

## Methods

### Ethics

The study was approved by the St Vincent's Hospital Human Research Ethics Committee (HREC 009.19) and all participants provided written informed consent.

### Study overview

The study collected long-term HR data from wearable devices and self-reported seizures from a mobile app to generate individual, personalised seizure forecasts based on seizure cycles, HR cycles and a combination of both. Each individual's forecast was initially trained using their first 10 reported seizures. Forecasts generated one likelihood per hour, and the projected hourly likelihood was averaged to also compute daily forecasts (one forecast per day instead of one forecast per hour). Forecasts were designed to run in a real-world mobile application, whereby hourly and daily likelihoods would be visualised (projected) for 60 days into the future. In the mobile app, risk forecasts were reissued weekly or after every reported seizure (whichever came first). To perform a blinded evaluation of forecast performance, each reissued forecast was stored and concatenated in chronological order to obtain the pseudo-prospective time series of seizure likelihood for the entire recording period. This pseudo-prospective likelihood was then assessed against the actual reported seizure times (excluding the initial training seizures).

All analyses were conducted in Python v3.7.

### Participants

Long-term HR data were recorded using wearable smartwatches (Fitbit, Fitbit Inc., USA) from 13 participants (6 males, mean age = 44 years, SD = 19 years) diagnosed with epilepsy and experiencing uncontrolled seizures. Participants were enrolled between October 2019 and January 2021 from an existing clinical study of long-term HR cycles.^25^ Participant were excluded if at least four months of monitoring data and 20 seizures had been recorded at the time of analyses. There were no exclusion criteria based on clinical or demographic features. Randomisation or blinding was not performed. Participants were instructed to wear smartwatches and report seizures using a smartphone app (Seer App, Seer Medical Pty Ltd, Australia). Participant characteristics are shown in Table 1.

**Table 1:** Participant characteristics.

ID	Sex	Age	Epilepsy	Syndrome/onset	Anti-seizure medication
**1**	F	36	Focal	Temporal	Lacosamide, Brivaracetam, Zonisamide, Losartan
**2**	M	27	Focal	Multi-focal	Levetiracetam, Carbamazepine, Lamotrigine, Perampane, Zonisamide
**3**	M	55	Focal	Temporal	Levetiracetam, Topiramate, Lamotrigine
**4**	F	31	Focal		Levetiracetam, Lacosamide
**5**	F	30	Generalised	JAE	Sodium Valproate, Lamotrigine, Perampanel
**6**	M	70	Focal	Temporal	
**7**	M	36	Focal	Temporo-parietal	Levetiracetam, Topiramate, Lamotrigine
**8**	F	55	Focal	Temporal	Topiramate, Lamotrigine, Carbamazepine
**9**	F	27	Focal	Temporal	Levetiracetam, Topiramate, Lacosamide
**10**	F	29	Focal	Multi-focal	Oxcarbazepine
**11**	M	36	Focal & Generalised	DEE	Lamotrigine, Clonazepam, Oxcarbazepine, Zonisamide
**12**	M	31	Focal	Temporal	Carbamazepine
**13**	F	69	Generalised	JAE	Sodium Valproate, Lamotrigine

Sex, age, epilepsy type, syndrome or onset location (if known) and anti-seizure medication types. JAE = Juvenile Absence Epilepsy, DEE = Developmental and Epileptic Encephalopathy.

### Training, testing and real-world evaluation data

The study used training and testing datasets, followed by an evaluation period using data collected prospectively after forecasting algorithms were developed (with participants blinded to their risk forecasts). The training dataset for each participant was taken from the start of recording until 10 seizures were recorded. The training cut-off time point was midnight on the day when the 10th seizure was reported. The testing dataset included continuous recordings and seizures reported from the training cut-off time point until 20 August 2021. The evaluation dataset included continuous recordings and seizures reported from 21 August 2021 until 5 April 2022. Forecasts were retrained after each reported seizure.

### Seizure forecasting

**Fig. 1:**
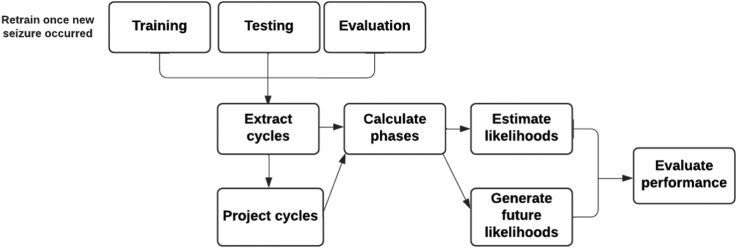
**A schematic diagram of the study design.** To train forecasting models, cycles are first extracted from HR and seizure diary data, converted to a continuous phase then the likelihood of a seizure with respect to each cycle phase is computed and combined (across both cycle periods and data types) into a single likelihood. During testing and prospective evaluation, extracted cycles are also projected into the future for a 60-day period. Projected cycles are used to compute future likelihoods in accordance with the likelihood model developed during training.

An overview of the forecasting pipeline is shown in Fig. 1. The training steps are: extract cycles, calculate phases, and estimate likelihoods. These steps are performed after each reported seizure (i.e., forecasts are retrained). The evaluation steps, performed for both retrospective testing and prospective evaluation, are: project cycles (using the results of cycle extraction), calculate phases, generate future likelihoods (using the results from likelihood estimation). Each element of the presented pipeline is outlined in the following subsections.

#### Cycle extraction

HR data were recorded at 0.2 Hz sampling rate and averaged over 5 min intervals. Missing HR data segments were set to a constant value at the mean HR in the training dataset. We had previously considered different interpolation approaches to fill gaps of missing heart rate data^25^; however, the constant mean was found not to alter cycle detection and was preferred for its simplicity. A continuous Morlet wavelet transform was then applied to the HR signal and significant peaks in the global wavelet power spectrum (using a time averaged significance test^28^) were considered significant HR cycle periods. Finally, HR cycles were derived by filtering HR signals (a zero-phase, second-order Butterworth bandpass filter) at the significant periodicities with a filter bandwidth of 30% of the central frequency (i.e., a cycle period of 8-days would us a filter bandpass from 5.6 days to 12.4 days).

Seizure cycles were extracted using the method presented in Karoly et al. (2021), whereby a range of patient-specific cycle periods were used to assess phase locking of self-reported seizures.^14^ Fixed underlying cycles with periods ranging from 1-day to 70-days (in-creased in 0.5-day increments) were applied to test the phase locking of seizure times. Only cycle periods of up to half the recording duration were considered, i.e., if the initial training period was 20-days then cycles of up to 10 days were considered. Therefore, longer cycles could be included as the amount of data increased.

The Hilbert transform was used to calculate the instantaneous phase of HR cycles. The instantaneous phase of seizure cycles is equal to the ratio of each time point to the cycle period (using a fixed sinusoid model). At each seizure onset time, the instantaneous phase was noted. The synchronization index (SI, also called the phase-locking value: PLV, or R-value) was used to quantify phase-locking of seizure times to different HR and seizure cycles. The SI is given by:

$$SI=\frac{1}{N}\sum_{n=1}^{N}\exp\left(i\theta_{n}\right)$$

where *θ*_*n*_ is the phase of the *nth* seizure with respect to an underlying cycle. The minimum SI threshold was set to 0.3. HR or seizure cycles with phase-locking above this threshold were selected to develop forecasts. A cut-off SI of 0.3 was chosen to reflect ‘moderate’ phase clustering.^9^

#### Estimate likelihoods

Seizure likelihood given the phase of each cycle was calculated as follows. The phase of one cycle period (–*π*, *π*) was divided into 18 equally spaced bins. The seizure likelihood given the phase of one cycle equals the number of seizures that occurred in the phase bin divided by the number of times the phase bin presented in the cycle. The seizure likelihoods, *p*_*i*_, in HR or seizure cycles were combined across different cycles, *i*, using a simple logit model. In this way, multiple cycles across different timescales were combined so a single estimate of seizure likelihood, *p*_*s*_ was obtained for each data type (HR and diary).

$$p_{s}=\frac{p}{1\;+\;p}$$

$$p=\prod_{i=1}^{n}{\left(\frac{p_{i}}{1\;-\;p_{i}}\right)}^{\frac{1}{n}}$$

In each forecast, two thresholds were set to classify the combined likelihood as either low, medium, or high-risk levels. The medium and high-risk cutoff thresholds were computed by optimizing two criteria:

1. seizures in high risk *>* seizures in medium risk *>* seizures in low risk
2. time spent in low risk *>* time spent in high risk

The second criterion was optimised after the first criterion; and, if no value could be found to satisfy the second criterion in conjunction with the first, then only the first criterion was used.

#### Cycle projection

The ‘Prophet’ (Facebook, CA, USA) model was used to project significant HR cycles to enable estimation of seizure risk for future time windows. Prophet is an open-source algorithm for automatically forecasting time series data, which is particularly useful for data that have cyclic trends, missing data, and outliers.^29^ In this study, the Prophet model was fit to HR data and projected for the next 60 days. The Prophet model is based on a piecewise-linear trend, various periodic changes, the effects of errors, and white noise error terms, which can be expressed as follows^30^:

$$y_{t}=g_{t}\;+\;s_{t}\;+\;h_{t}\;+\;\varepsilon_{t}$$

Where $g_{t}$ represents non-periodic changes in time series at the time point *t*, $s_{t}$ represents periodic changes, with yearly, weekly, and daily cycles included by default, $h_{t}$ represents the effects of holidays (not included by default), and $\varepsilon_{t}$ is an error term. Cyclic effects are provided using Fourier series of the relevant periods, which can be represented as follows^31^:

$$s_{t}=\sum_{n=1}^{N}a_{n}\;\cos\left(\frac{2\prod nt}{P}\right)+b_{n}\;\sin\left(\frac{2\prod nt}{P}\right)$$

where P represents a regular period. The current study used the default Prophet model settings (daily, weekly, and yearly rhythms) with additional rhythms added for each HR cycle. Regional holiday effects were not included.

Seizure cycles were projected forwards in time using a fixed sinusoid model, with one sinusoid for each seizure cycle frequency (i.e., each cycle with a SI value above 0.3).

#### Generate future likelihoods

The instantaneous phase of the projected cycles (HR and seizure cycles) was calculated as described in the section, ‘Cycle Extraction’. The projected phases were used to estimate seizure likelihoods using the likelihood models presented in the section, ‘Estimate Likelihoods’. Seizure likelihoods were generated every hour and projected into the future. Forecasting algorithms were retrained weekly, or after each seizure occurred, resulting in regeneration of the projected likelihoods. We also assessed performance using a daily forecast resolution, by averaging the projected likelihoods across each 24-h period.

#### Combine likelihoods

The combined forecast was developed based on likelihoods generated by a HR forecast and a diary forecast. The logit model was used to combine two seizure likelihoods for each time point. The optimal forecast was chosen for each participant by selecting the forecast with the highest area under the curve (AUC) of the receiver operating curve (ROC). The ROC was defined here as the plot of the time spent in high-risk versus the true positive rate, as the high-risk threshold was varied.

### Statistics

To test whether forecasting models performed significantly better than chance, the surrogate approach was applied.^8^^,^^32^ We randomly shuffled seizure occurrence times for each participant in the testing or evaluation period and recalculated the AUC value. This procedure was repeated 200 times and the distribution of AUC values was recorded. The number of simulated seizure times was derived from all seizure hours (seizure days for daily resolution forecasts) of each participant in the testing or evaluation periods. Forecasts were considered to show improvement over chance (IoC) if the AUC was greater than 95% of all simulated AUC values (p < 0.05).

A one-way ANOVA test was used to assess the performances of different forecasts at a group level. To measure whether performance improved over time, the Wilcoxon signed rank test was used to test AUC of participants in testing and evaluation periods.

The Brier Skill Score (BSS) was also used to assess the accuracy of forecasts compared to a random, reference forecast (BSS = 1 for perfect forecast; BSS = 0 for no improvement over a random predictor). The BSS provides complementary information to the AUC, as it does not require a seizure prediction threshold, but rather assesses how closely a continuous, probabilistic forecast aligns with the observed occurrence of seizures.^11^

### Role of funders

None of the funders had a role in study design, data collection, data analyses, interpretation, or writing of the report. Seer Medical provided the cloud platform and mobile app for secure data storage, data access, and algorithm deployment.

## Results

### Data

Table 2 shows an overview of the collected data. The mean period of recording was 562 days (SD = 262 days). On average, 125 (SD = 108) seizures were reported per person. The training dataset for each participant was taken from the start of recording (M = 96 days, SD = 91 days) until 10 seizures were recorded. The training cut-off time point was midnight on the day when the 10th seizure was reported (the 10th, 11th, and 12th seizures in P3, and the 10th and 11th seizures in P12 occurred on the same day). The testing dataset included continuous recordings (M = 350 days, SD = 167 days) and seizures (M = 95, SD = 94) reported from the training cut-off time point until 20 August 2021. The average proportion of continuous HR data collected from smartwatches during the testing periods was 82.4% (ranging from 98% to 45%). Data dropout rates were similar in the prospective evaluation period.

**Table 2:** Data characteristics.

ID	Seizure counts		Recording duration (days)		Dropout %
	Training	Testing	Training	Testing	
**1**	10	103 (40)	57	490 (228)	3 (9)
**2**	10	75	36	183	20
**3**	12	201	46	438	14
**4**	10	20 (16)	321	304 (272)	2 (22)
**5**	10	161	5	267	37
**6**	10	14 (6)	228	385 (273)	19 (24)
**7**	10	42	70	346	54
**8**	10	23	105	481	4
**9**	10	240 (103)	48	586 (228)	27 (21)
**10**	10	61 (85)	73	461 (232)	23 (10)
**11**	10	276	14	481	9
**12**	11	10 (17)	161	88 (272)	12 (15)
**13**	10	4	90	35	4
		M: 95 (45) SD: 94 (40)	M: 96 SD: 91	M: 350 (251) SD: 167 (24)	

Number of seizures, recording duration in days and proportion of missing HR data (%). Values in brackets reflect the evaluation period for 6 participants.

For evaluation, 7 participants’ (3 female) data were lost to follow up. Two participants reported that they had become seizure free (although wearable data continued), one moved overseas, and the remaining four ceased data collection (both HR and event reporting) and could not be contacted. For the remaining 6 participants, the evaluation period was 251 days on average (SD = 24 days), with an average of 45 reported seizures (SD = 40).

### Projection of HR cycles

**Fig. 2:**
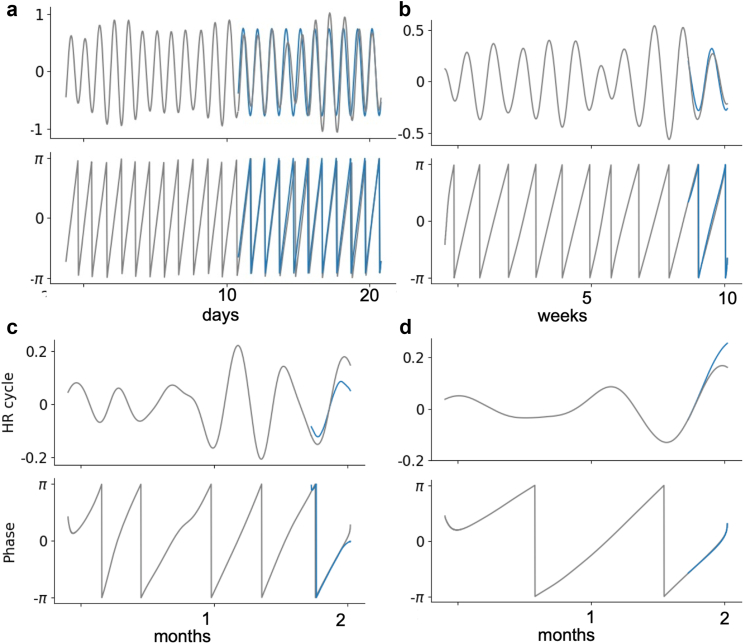
**Significant HR cycles projected using the Prophet model for the testing dataset of P12**. Examples of daily, weekly, 2-weekly and monthly HR cycle projections are shown in subplots a-d. In each subplot, the grey lines are recorded HR cycles (top panel) and instantaneous phases (bottom panel) of cycles. The blue lines represent the projected HR cycles and corresponding phases.

The most common detected HR cycles across participants were daily (85%), weekly (38%), 2-weekly (23%), and monthly (38%) cycles (Supplementary Fig. S1 and Table S1). Fig. 2 shows examples of projected daily, weekly, 2-weekly, and monthly HR cycles using Prophet models for P12 in the testing dataset. The accuracy of the Prophet model for all HR cycle projections across participants is shown in Supplementary Tables S2 and S3.

### Seizure forecasting performance

In the testing period, seizures were forecast above chance in 9/13 and 11/13 participants based on hourly and daily resolutions, respectively. The performance of the best forecast (diary, HR, combined) for each participant is shown in Table 3. For hourly forecasts, the best performance was from diary-based forecasts (used for 6 out of the 9 significant forecasts), while daily forecasts were more distributed (5 HR and 6 diary). For participants with above-chance performance, the mean hourly sensitivity, time in high-risk, and AUC of the best forecasts were 61%, 27%, and 0.73. The mean BSS was 0.05 and 0.23 for hourly and daily resolution forecasts, respectively. In the prospective evaluation period, 4/6 and 5/6 participants showed above-chance performance based on hourly and daily resolutions. For participants with above-chance performance in evaluation, the mean hourly sensitivity, time in high-risk and AUC were 56%, 18% and 0.77. The AUC values of the best forecasts for the 6 participants in prospective evaluation were significantly increased (Wilcoxon signed rank test, p = 0.031) compared to the testing period.

**Table 3:** Performance of forecasts for hourly and daily resolution.

ID	Hourly forecasts					Daily forecasts		
	Type	AUC	BSS	Sens (%)	TiH (%)	Type	AUC	BSS
**1**	D (−)	0.67 (0.69)	0.02 (0.03)	60 (53)	37 (27)	D (D)	0.63 (0.65)	0.08 (0.19)
**2**	C	0.83	0.12	58	15	HR	0.96	0.60
**3**	D	0.68	0.04	62	35	D	0.69	0.19
**4**	D (D)	0.83 (0.83)	0.05 (0.04)	60 (56)	11 (6)	D (C)	0.69 (0.68)	0.10 (0.09)
**5**	D	0.68	0.06	60	33	HR	0.63	0.12
**6**^a^	− (−)	0.57 (0.79)	0.01 (0)	29 (67)	20 (8)	D (HR)	0.68 (0.98)	0.04 (0.05)
**7**	D	0.76	0.04	75	30	D	0.66	0.10
**8**	–	0.61	0.01	4	6	HR	0.68	0.09
**9**	C (C)	0.71 (0.78)	0.02 (0.02)	66 (85)	35 (37)	− (−)	0.50 (0.50)	0 (0)
**10**	HR (C)	0.82 (0.82)	0.06 (0.09)	56 (59)	11 (16)	HR (HR)	0.87 (0.89)	0.40 (0.48)
**11**	D	0.62	0.04	53	34	D	0.56	0.02
**12**	- (D)	0.50 (0.66)	0 (0.01)	20 (24)	13 (12)	- (D)	0.56 (0.71)	0.03 (0.20)
**13**^a^	–	0.93	0	100	10	HR	1.0	0.74
	IoC: 9/13 (4/6)	M: 0.73 (0.77)	M: 0.05 (0.04)	M: 56 (52)	M: 22 (14)	IoC: 11/13 (5/6)	M: 0.73 (0.78)	M: 0.23 (0.20)

Type indicates the data that provided the best performance based on AUC (D = diary, HR = heart rate, C = combined). Forecasts that did not perform significantly above chance (p < 0.05 using surrogate analysis) are shown as a dash. IoC=Improvement over chance, summarises the number of people with above chance performance. Sens = Sensitiviy and TiH=Time-in-high risk, are reported as percentages for hourly forecast resolutions. BSS=Brier Skill Score. Values in brackets are for the prospective evaluation periods (P1, P4, P6, P9, P10, P12). Mean values were computed across participants with above chance performance only.

a Participants reported fewer than 10 seizures during testing or evaluation periods, which may bias performance.

**Fig. 3:**
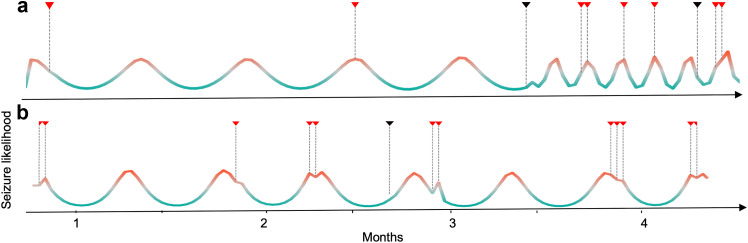
**Example daily risk forecasts for two participants. a)** Heart rate forecast for P10 **b)** Diary forecast for P1. The coloured line shows forecast seizure likelihood. Triangles show reported seizure occurrence, where red indicates seizure occurred in high risk and black indicates seizure occurred in low or moderate risk.

Fig. 3 shows example forecasts and seizure times for two participants (P1 and P10) daily forecasts, using heart rate and diary data. Daily forecast performance was similar overall to hourly forecasts, with mean AUC of 0.73 and 0.78 in testing and evaluation periods, respectively. Two participants’ (P2 and P5) best forecast method was different using daily, compared to hourly forecasts.

### Forecast comparison

This section compares forecasting performance to the time-of-day model as a baseline. The time-of-day model used only the clock time to output an hourly likelihood based on the prior distribution of seizures with respect to time-of-day. In the testing period, the time-of-day model had average AUC across all participants of 0.66 (Supplementary Table S5), compared to average AUC of 0.71 using cycle-based forecasts (Supplementary Table S4). In the prospective evaluation, the time-of-day model had average AUC across all participants of 0.69 (Supplementary Table S5), compared to 0.76 using cycle-based forecasts (Supplementary Table S4). The cycle-based models were marginally better than the time-of-day models at the hourly scale (p = 0.047 in both testing and evaluation periods using the Wilcoxon signed rank test).

The mean AUC of the daily forecasts across all participants was 0.70 in the testing period and 0.74 in the prospective evaluation period (Supplementary Table S4). Unsurprisingly, when the time-of-day models were used to produce a daily forecast, the resulting forecasts showed chance level performance, with average AUC of 0.51 and 0.49 across all participants in the testing and evaluation periods, respectively (Supplementary Table S5). The time-of-day and cycle-based models were significantly different at the daily resolution (Wilcoxon signed rank test, p < 0.001 in both testing and evaluation periods).

**Fig. 4:**
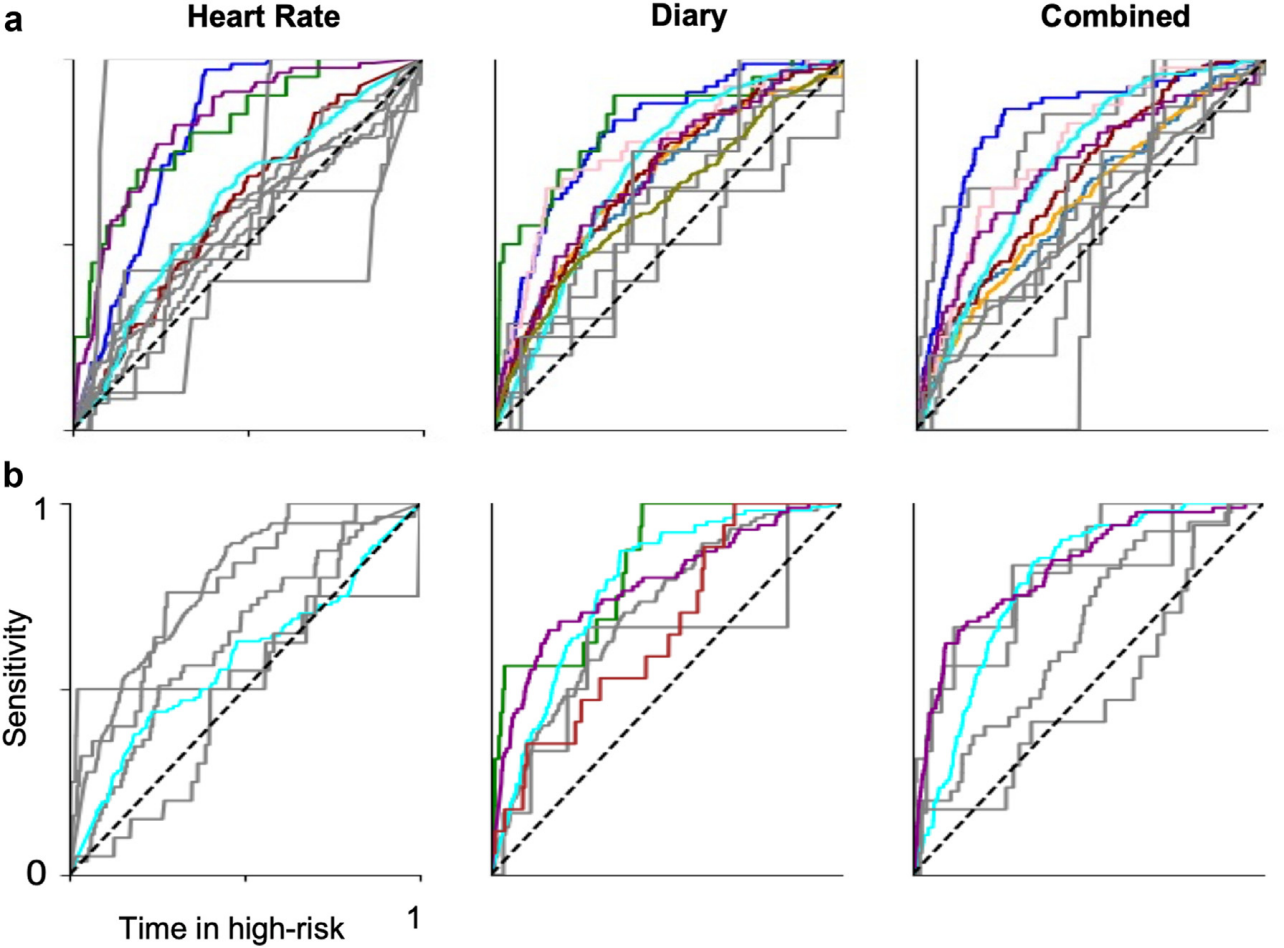
**ROC curves of HR, diary and combined hourly forecasts.** (a) ROC curves for 13 participants in the HR (left), diary (middle) and combined (right) forecasts using testing data. (b) ROC curves for 6 participants in the HR (left), diary (middle) and combined (right) forecasts using prospective data.

The contribution of HR and diary inputs was also compared. Fig. 4 shows the ROC curves for HR, diary, and combined forecasts in the testing (Fig. 4a) and evaluation (Fig. 4b) periods. During testing, the hourly AUC values of HR, diary and combined forecasts were not significantly different at a group level (One-way ANOVA, F = 0.18, p = 0.83). During prospective evaluation, the mean hourly AUC across all participants for HR, diary, and combined forecasts (0.64, 0.73, and 0.73) were higher than the test period (0.64, 0.67, and 0.65, Supplementary Table S4).

## Discussion

This study presents a prospective framework for non-invasive, self-reported seizure forecasting based on well-established cycles of multimodal data, which can readily be generalized for different signal types. During testing, most of the cohort showed performance above chance with a mean AUC of 0.73 (Table 3). The forecasts were evaluated in a prospective setting, giving a mean AUC of 0.77 with 4/6 of cohort showing performance above chance (Table 3). Prospective evaluation demonstrated that forecasting was robust in a blinded, real-world application. Forecasting performance was similar with a daily forecast resolution, with 11/13 and 5/6 participants showing improvement over chance (mean AUC of 0.73 and 0.78) during testing and prospective evaluation, respectively. Forecast performance was similar to the state-of-the-art retrospective forecasting studies in cohorts with self-reported seizures.,^8^^,^^14^^,^^24^ highlighting the potential for clinical translation of non-invasive, real-world forecasting.

### Forecasting performance

Previous studies used physiological data recorded from wearable devices to forecast seizures with varying success.^2^^,^^22^^,^^24^ Meisel et al. used electrodermal activity, body temperature, blood volume pulse, and actigraphy from a wristband sensor to predict seizures with better-than-chance predictability in 43% of the participants.^22^ Nasseri et al. used accelerometry, blood volume pulse, electrodermal activity, and temperature to achieve a mean AUC of 0.75 with 5/6 of participants showing performances better than chance.^2^ Stirling et al. applied HR, sleep, and step counts to forecast seizures with a mean AUC of 0.74 and all 11 participants showing performances better than chance.^24^ The current work achieved comparable mean AUC values to these previous studies using cycle-based models (combining cyclic likelihoods with logit models). These models used less parameters and less memory to implement compared to black-box machine learning or deep learning algorithms used in previous research. A similar forecasting algorithm used multidien cycles recorded intracranially and reported median daily AUC of 0.70 and BSS of 0.13 for self-reported seizures,^8^ showing similar performance as the current, non-invasive approach. Moreover, the performance of the current study was validated on blinded, prospective data, which is an important step towards clinical applications.

The current study used the Prophet model to project HR cycles forward in time to estimate future seizure likelihood, thus enabling prospective evaluation of seizure risk forecasting. The Prophet model has been widely used in predicting time-series data,^30^^,^^31^^,^^33^^,^^34^ and the current results showed that HR cycle projections were accurate over weeks (Fig. 2, Supplementary Tables S2 and S3). The Prophet model could also be applied to multiday cycles recorded from other wearable or implantable device used in seizure forecasting.

Hourly forecasts did not show a clear increase in performance compared to time-of-day models, which simply used the likelihood of seizures with respect to clock time to estimate future seizure risk. This is partly due to the strong contribution of circadian cycles to the risk of seizure occurrence,^35^ although reporting bias also contributes to circadian cycles of self-reported seizures.^27^ Nevertheless, electrographic seizures also show a circadian profile and the utility of time-of-day to track seizure risk should not be ignored.^11^ Multidien rhythms provide a stronger contribution to seizure risk forecasts than circadian cycles for many people.^8^^,^^16^ The current results also showed the performance of cycle-based forecasts at a daily resolution was significantly higher than time-of-day forecasts (which are equivalent to chance level on a daily timescale), highlighting the role multidien cycles play when tracking seizure risk over longer timescales. In line with this, the mean BSS was 0.23 for daily resolution forecasts, but just 0.05 for hourly resolution (Table 3), which is similar to previous studies,^8^^,^^11^ suggesting that slower timescale, or ‘smoother’ forecasts provide a better representation of the underlying probabilistic dynamics. Practically, users' preferences regarding hourly or daily forecasts are likely to vary between individuals and depend on their intended use of risk forecasts in addition to other factors, like seizure frequency.

On average, 10 training seizures and 96 days of HR data were used to develop forecasting models in the current study (Table 2). However, the performance for some participants is expected to improve as longer data and more seizures become available. For instance, the four participants who showed chance-level performance (P6, P8, P12, P13, Table 3), all had a small number of test seizures (M = 12, SD = 8). We chose 10 training seizures to reflect forecasters that could be rapidly deployed in the real world. For most practical clinical applications, users of a seizure risk forecasting app are unlikely to wait months to years to receive their first forecast (the maximum training period in this study was 321 days). However, as forecasts were retrained after each reported seizure, the amount of training data continually increases, enabling forecasting performance to improve over time. In a real-world application, users would only become unblinded to their forecast if/when above-chance performance was achieved, which in practice may require more than 10 reported seizures for training.

### Multimodal data comparison

At a group-level, there was no significant difference between the AUC of diary, HR, or combined forecasts (Fig. 4, One-way ANOVA, F = 0.14, p = 0.86); however, the optimal choice of forecasts was subject-specific (Table 3). HR data contributed to seizure forecasting for three out of nine people with significant AUC performance in testing, and two out of four in evaluation. In some participants, such as P10 (Fig. 3a), the AUC performance was greatly increased using an HR forecast (0.82) than a diary forecast (0.69). Only two out of nine people with significant forecasts during testing used combined signals, suggesting increased multimodality is not critical; however, collecting multimodal signals allows greater flexibility to update models. For instance, during prospective evaluation, the best-performing forecast changed in 2/6 of participants (P10 and P12). In P10, the best-performing method changed from an HR forecast to a combined (HR + diary) forecast, highlighting the importance of using multimodal data. In P12, the forecast performance went from chance level in the testing period (AUC = 0.50) to above-chance performance (AUC = 0.66) in the evaluation period, suggesting the importance of continuing to evaluate forecasts with long-term, prospective data.

HR and seizure cycles showed similar distributions in testing datasets. HR forecasts used daily HR cycles in 11/13 of participants, weekly cycles in 5/13, 2-week cycles in 3/13, and monthly cycles in 2/13 (Supplementary Table S1). In comparison, diary forecasts used daily seizure cycles in 11/13 of participants, weekly cycles in 8/13, 2-week cycles in 4/13, and monthly cycles in 3/13 (Supplementary Table S1). Moreover, the current study provides insight into the changes of significant cycles over time. The results showed that significant HR and seizure cycles changed in 4/6 and 6/6 of participants in prospective periods compared to testing periods (Supplementary Table S1), which may contribute to the changes in AUC values. For participants with increased AUC values (P1, P12) in HR forecasts, more HR cycles were involved to develop forecasts. For all 6 participants with increased AUC values using diary forecasts, the number of seizure cycles was decreased, demonstrating the importance of exploring significant seizure cycles with long-term data.

### Caveats and limitations

A limitation of this study is HR data were recorded from a wearable device, which is prone to artifacts and noise. However, wearable devices for HR data are becoming increasingly advanced in medical applications, with Food and Drug Administration (FDA) approval for management of cardiac conditions. Participant adherence also introduces noise, as discontinuous HR data in some participants may generate low performance models. The current study did not appear to show a relationship between the proportion of HR dropout (Table 2) and forecast performance, although the cohort was too small to fully evaluate this possibility.

The small number of total participants (n = 13) and number who were prospectively evaluated (6/13) is a major limitation of this study, and as such, the results can only be interpreted as a preliminary proof-of-principle that real-time, prospective forecasting of self-reported seizures is feasible. The small cohort restrict the ability to generalise findings to the wider population of people with epilepsy, and robust analysis of clinical or demographic factors that might re-late to forecasting performance was not possible. However, the cohort consists of a clinically representative snapshot of adults with epilepsy (10 focal, including 6 TLE), showing a balance of genders and ages. Participants’ who were lost to follow up (3 female, 4 male) included all three generalised epilepsy cases. However, there was unlikely to be a performance bias resulting from the participants lost to follow up, since participants were blinded to risk forecasts. Participants were on a range of anti-seizure medications during the study, and continued standard clinical care, including possible medication changes, which may have affected their seizure or HR cycles.^36^ Future work should investigate the effect of medications on forecast performance, noting that in real world applications, seizure risk forecasts need to be compatible with ongoing clinical management.

The current study was based on participants’ self-reported seizures which provide a biased representation of seizure burden. Therefore, risk forecasts represent the likelihood of a reported seizure, rather than the likelihood of an electrographic seizure. The limitations of this approach include the possibility of underestimating the risk of certain event types, or nocturnal events, which are subject to different reporting biases. Nevertheless, forecasting reported seizure risk does not negate all clinical utility. Indeed, self-reported seizures remain the standard metric used in both clinical trials and clinical management for epilepsy. Furthermore, previous work suggests that self-reported seizures align to the same underlying cycle phase as electrographic seizures.^16^ Other studies showed that seizure cycles measured from self-reported events were consistent with cycles extracted from electrographic seizures for around half a cohort with chronic implanted EEG,^14^ and self-reported seizure cycles have been found to align with epileptic activity captured on video-EEG.^37^ Seizure detection methods that capture electrographic seizures are required to fully validate risk forecasting algorithms. Similarly, devices that record continuous EEG enable true estimation of underlying epileptic rhythms, which have enabled the most accurate estimation of seizure risk cycles to-date.^8^^,^^12^ Future work will focus on validating the presented forecasting system using seizures recorded from EEG, including cohorts implanted with sub-scalp EEG and responsive neurostimulation devices.

Finally, future work will validate performance in a larger cohort to explore additional clinical, demographic, and technical factors that could influence forecast accuracy. For instance, age,^38^ anti-seizure medications^36^ and seizure type or onset lobe^16^^,^^27^ may all influence underlying multiday seizure cycles which could impact the forecasting algorithms deployed here. Similarly, several methodological choices were made in this study that require a larger cohort to justify or further optimise. The models used for cycle extraction, including the SI threshold, and cycle projection may all be improved with further parameter optimisation or through comparison against alternative methods. It is important to note that any methods evaluated for seizure forecasting must be compatible with prospective or real-time implementation. Practical considerations include synchronising multimodal data (where accurate timestamps are critically important), dealing with missing data, computational requirements and the costs and technical infrastructure required to store data (including past and future forecasts) and update forecasts at scale (i.e., increasing computational requirements as more users download the device). Recent developments in cloud computing for medical data have vastly reduced computational bottlenecks, and these costs are unlikely to limit all but the most complex and data intensive forecasting models. However, the most fundamental hurdle in moving from retrospective to prospective validation is ensuring reliability and security, which requires a highly regulated database and software environment, with ongoing testing and maintenance that is not sustainable within most research environments. Future challenges include incorporating different wearable device types (with different data specifications) and supporting the diverse technology access levels and literacy of users (i.e., are smartphones or network connectivity required). Most importantly, the next iteration of unblinded seizure risk forecasting technology must involve users in the design process to understand and optimise the user experience for the broader epilepsy community.

## Contributors

All authors read and approved the final version of the manuscript.

WX led the data analysis, code and figure generation, writing and editing of the text.

PJK conceived of and led the study, contributed to conception of ideas, and editing of the text ESN, RES and DP contributed to study design, data generation and analysis, code and figure generation, and editing of the text.

TK, MJC, DRF, PFV, BHB, MPR contributed to study design, conception of ideas and editing of the text.

WX, RES and DEP verified the underlying data.

## Data sharing statement

Excluding participants who did not consent to share their data publicly, deidentified data are available on Figshare (as DOI 10.26188/23206445).

## Declaration of interests

Dr. Brinkmann reports grants from Epilepsy Foundation of America, My Seizure Gauge, and National Institutes of Health, during the conduct of the study; other from Seer Medical, UNEEG Medical and Medtronic, outside the submitted work, personal fees from Otsuka Pharmaceuticals, outside the submitted work, travel support from ICTALS, American Epilepsy Society, and ICCN. In addition, Dr Brinkmann has patents pending, outside the submitted work.

Dr. Stirling reports grants from Australian Government Research Training Program Scholarship, Epilepsy Foundation of America, and Australian Government BioMedTech Horizons Program, during the conduct of the study.

Dr. Karoly reports grants from National Health and Medical Research Council (NHMRC), and Australian Government BioMedTech Horizons Program, during the conduct of the study; personal fees and other from Seer Medical, outside the submitted work; Travel support from The University of Melbourne, NHMRC, International League Against Epilepsy; In addition, Dr. Karoly has a patent Methods and Systems of Seizure Forecasting issued.

Dr. Cook reports grants from Epilepsy Foundation of America, during the conduct of the study; personal fees and other from Seer Medical Australia, Epi Minder and Cerebral Therapeutics outside the submitted work; stock or stock options from Seer Medical Pty and Epi Minder; payment from expert testimony from the Federal Court of Australia; support for travel and meetings from the University of Melbourne; grants from NHMRC and Australian Research Council, outside the submitted work. In addition, Dr. Cook has a patent Methods and Systems of Seizure Forecasting issued.

Dr. Nurse reports grants from Epilepsy Foundation of America, grants from MTPConnect, during the conduct of the study; and is an employee of Seer Medical.

Dr. Freestone reports grants from Epilepsy Foundation USA and is an employee of Seer Medical. In addition, Dr. Freestone has a patent Methods and Systems of Seizure Forecasting issued.

Dr. Richardson reports grants from Epilepsy Foundation of America, during the conduct of the study; personal fees from UNEEG Medical, outside the submitted work; grants from the Medical Research Council, NIH, Epilepsy Research UK, GW Pharma, Maudsley Charity, European Commission, outside the submitted work.

Dr. Viana reports grants from Epilepsy Foundation of America, during the conduct of the study; personal fees from UNEEG Medical and Eisai Pharmaceuticals, outside the submitted work; and travel support from UNEEG Medical.

Dr Payne is an employee of Seer Medical.

All other authors have no interests to disclose.

## References

[1] Janse S.A., Dumanis S.B., Huwig T. (2019). Patient and caregiver preferences for the potential benefits and risks of a seizure forecasting device: a best–worst scaling. Epilepsy Behav.

[2] Nasseri M., Pal Attia T., Joseph B. (2021). Ambulatory seizure forecasting with a wrist-worn device using long-short term memory deep learning. Sci Rep.

[3] Litt B., Esteller R., Echauz J. (2001). Epileptic seizures may begin hours in advance of clinical onset: a report of five patients. Neuron.

[4] Mormann F., Kreuz T., Andrzejak R.G., Lehnertz K., Elger C.E. (2003). Epileptic seizures are preceded by a decrease in synchronization. Epilepsy Res.

[5] Lange H.H., Lieb J.P., Engel J., Crandall P.H. (1983). Temporo-spatial patterns of pre-ictal spike activity in human temporal lobe epilepsy. Electroencephalogr Clin Neurophysiol.

[6] Iasemidis L.D., Sackellares J.C., Zaveri H.P., Williams W.J. (1990). Phase space topography and the Lyapunov exponent of electrocorticograms in partial seizures. Brain Topogr.

[7] Mormann F., Andrzejak R.G., Elger C.E., Lehnertz K. (2007). Seizure prediction: the long and winding road. Brain.

[8] Proix T., Truccolo W., Leguia M.G. (2021). Forecasting seizure risk in adults with focal epilepsy: a development and validation study. Lancet Neurol.

[9] Karoly P.J., Rao V.R., Gregg N.M (2021). Cycles in epilepsy. Nat Rev Neurol.

[10] Baud M.O., Proix T., Gregg N.M. (2022). Seizure forecasting: bifurcations in the long and winding road. Epilepsia.

[11] Karoly P.J., Ung H., Grayden D.B. (2017). The circadian profile of epilepsy improves seizure forecasting. Brain.

[12] Maturana M.I., Meisel C., Dell K. (2020). Critical slowing down as a biomarker for seizure susceptibility. Nat Commun.

[13] Baud M.O., Kleen J.K., Mirro E.A. (2018). Multi-day rhythms modulate seizure risk in epilepsy. Nat Commun.

[14] Karoly P.J., Cook M.J., Maturana M. (2020). Forecasting cycles of seizure likelihood. Epilepsia.

[15] Elger C.E., Hoppe C. (2018). Diagnostic challenges in epilepsy: seizure under-reporting and seizure detection. Lancet Neurol.

[16] Leguia M.G., Andrzejak R.G., Rummel C. (2021). Seizure cycles in focal epilepsy. JAMA Neurol.

[17] Brinkmann B.H., Karoly P.J., Nurse E.S. (2021). Seizure diaries and forecasting with wearables: epilepsy monitoring outside the clinic. Front Neurol.

[18] Stirling R.E., Maturana M.I., Karoly P.J. (2021). Seizure forecasting using a novel sub-scalp ultra-long term EEG monitoring system. Front Neurol.

[19] Pal Attia T., Viana P.F., Nasseri M. (2022). Seizure forecasting using minimally invasive, ultra-long-term subcutaneous EEG: generalizable cross-patient models. Epilepsia.

[20] Viana P.F., Pal Attia T., Nasseri M. (2022). Seizure forecasting using minimally invasive, ultra-long-term subcutaneous electroencephalography: individualized intrapatient models. Epilepsia.

[21] Beniczky S., Karoly P., Nurse E., Ryvlin P., Cook M. (2021). Machine learning and wearable devices of the future. Epilepsia.

[22] Meisel C., El Atrache R., Jackson M. (2020). Machine learning from wristband sensor data for wearable, noninvasive seizure forecasting. Epilepsia.

[23] Billeci L., Marino D., Insana L., Vatti G., Varanini M. (2018). Patient-specific seizure prediction based on heart rate variability and recurrence quantification analysis. PLoS One.

[24] Stirling R.E., Grayden D.B., D’Souza W. (2021). Forecasting seizure likelihood with wearable technology. Front Neurol.

[25] Karoly P.J., Stirling R.E., Freestone D.R. (2021). Multiday cycles of heart rate are associated with seizure likelihood: an observational cohort study. EBioMedicine.

[26] Gregg N.M., Pal Attia T., Nasseri M. (2023). Seizure occurrence is linked to multiday cycles in diverse physiological signals. Epilepsia.

[27] Karoly P.J., Goldenholz D.M., Freestone D.R. (2018). Circadian and circaseptan rhythms in human epilepsy: a retrospective cohort study. Lancet Neurol.

[28] Torrence C., Compo G.P. (1998). A practical guide to wavelet analysis. Bull Am Meteorol Soc.

[29] Hyndman R., Athanasopoulos G. (2018).

[30] Samal K.K.R., Babu K.S., Das S.K., Acharaya A. (2019). Proc 2019 Int. Conf. Inf. Technol. Comput. Commun.

[31] Borowik G., Wawrzyniak Z.M., Cichosz P. (2018). 2018 26th International Conference on Systems Engineering (ICSEng) vols. 1–10.

[32] Andrzejak R.G., Mormann F., Kreuz T. (2003). Testing the null hypothesis of the nonexistence of a preseizure state. Phys Rev E - Stat Nonlinear Soft Matter Phys.

[33] Aditya Satrio C.B., Darmawan W., Nadia B.U., Hanafiah N. (2021). Time series analysis and forecasting of coronavirus disease in Indonesia using ARIMA model and PROPHET. Procedia Comput Sci.

[34] Toharudin T., Pontoh R.S., Caraka R.E., Zahroh S., Lee Y., Chen R.C. (2023). Employing long short-term memory and Facebook prophet model in air temperature forecasting. Commun Stat Simulat Comput.

[35] Khan S., Nobili L., Khatami R. (2018). Circadian rhythm and epilepsy. Lancet Neurol.

[36] Halimeh M., Yang Y., Sheehan T. (2022). Wearable device assessments of antiseizure medication effects on diurnal patterns of electrodermal activity, heart rate, and heart rate variability. Epilepsy Behav.

[37] Karoly P.J., Eden D., Nurse E.S. (2021). Cycles of self-reported seizure likelihood correspond to yield of diagnostic epilepsy monitoring. Epilepsia.

[38] Wang E.T., Vannucci M., Haneef Z., Moss R., Rao V.R., Chiang S. (2022). A Bayesian switching linear dynamical system for estimating seizure chronotypes. Proc Natl Acad Sci USA.

