# Supplementary material for: Forecasting seizure likelihood from cycles of self-reported events and heart rate: a prospective pilot study
Source: eBioMedicine. 2023 Jun 16;93:104656. doi: 10.1016/j.ebiom.2023.104656 (PMC10300292; doi:10.1016/j.ebiom.2023.104656)
Supplement: Supplementary Figure S1 and Tables S1–S6 [file mmc1.docx]

**Supplement**

**
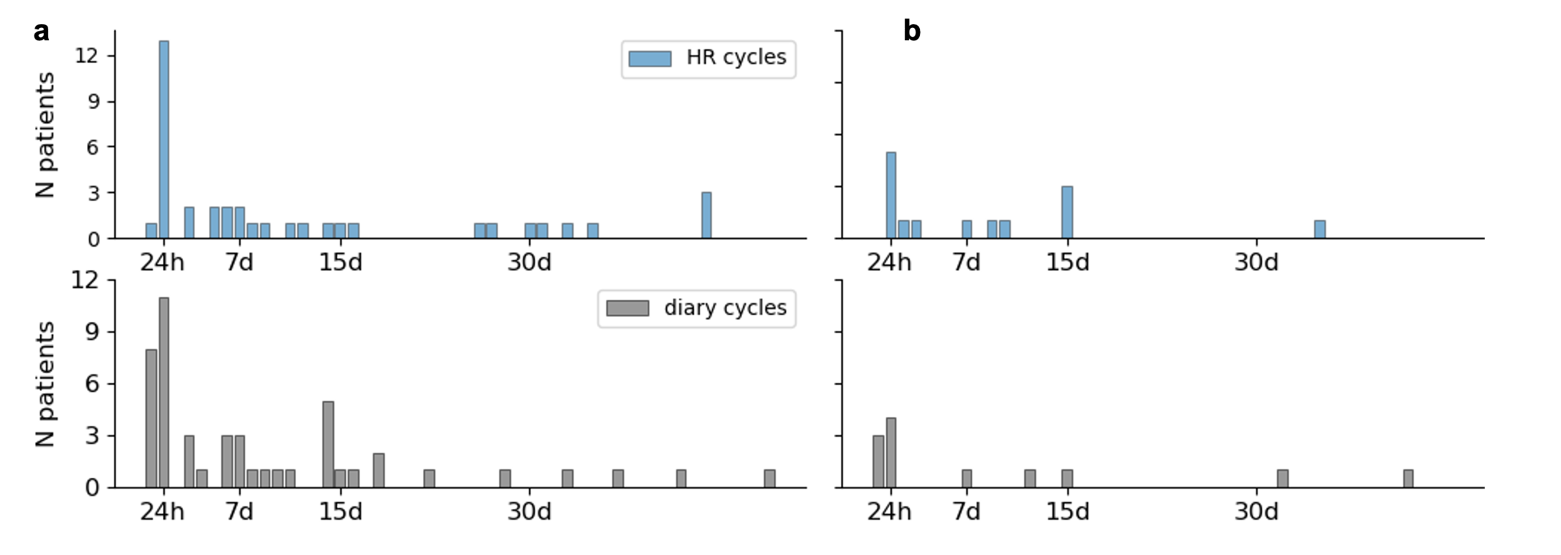
**

**Supplementary Figure 1. Significant HR and diary cycles used in the forecasts.** (a) Training and testing dataset; (b) Evaluation dataset

**Supplementary Table 1. Significant HR and diary cycles in the forecasts for each patient.**

| **ID** | **Heart rate cycles** | | **Diary cycles** | |
| --- | --- | --- | --- | --- |
|  | **Training and testing** | **Evaluation** | **Training and testing** | **Evaluation** |
| **1** | 24h, 15d, 27.5d | 24h, 10.5d, 15d | 24h, 7d, 14.5d | 24h, 15.5d |
| **2** | 24h |  | 12h, 24h, 6.5d, 15.5d |  |
| **3** | 24h, 6d, 11d, 26.5d, 35d, 44.5d |  | 14.5d, 18d, 22d, 28.5d |  |
| **4** | 24h, 3d, 7d, 44d, 92d, 104d | 24h, 2.4d, 7d, 100d, 175d | 12h, 24h, 3d, 7d | 12h, 24h, 7d |
| **5** | 24h |  | 6h, 24h, 18.5d |  |
| **6** | 12h, 1.8d, 3.4d, 6.5d, 9.5d, 16d | 3.4d, 9.5d, 15.5d, 52d | 12h, 14.5d, 42d, 52d | 12h, 42.5d |
| **7** | 24h, 5d, 31d |  | 6h, 24h, 3.5d, 11.5d |  |
| **8** | 24h |  | 24h, 8.5d, 10.5d, 14.5d, 37d |  |
| **9** | 24h, 79d | 24h, 128d | 12h, 24h, 7.5d | 24h |
| **10** | 24h, 30d | 24h, 110d, 185d | 12h, 24h, 5.5d, 33.5d | 12h, 24h |
| **11** | 24h, 5d, 8.5d, 12d, 44d |  | 18h, 24h, 3d |  |
| **12** | 7d, 14.5d, 33d | 24h, 15d, 35d | 24h, 6d, 16d, 49.5d | 12d, 32d |
| **13** | 24h |  | 24h, 6.5d, 9d, 14d |  |
| **Daily** | 85% | 83% | 85% | 67% |
| **Weekly** | 38% | 33% | 62% | 17% |
| **2-weekly** | 23% | 50% | 31% | 17% |
| **Monthly** | 38% | 17% | 38% | 33% |

Supplementary Tables 2 and 3 show the accuracy of the Prophet model for HR cycle projection. The R-squared score, also known as the coefficient of determination, is computed as the proportion of variance in the HR signal that is explained by the model. The mean squared error (MSE) is the difference between the model projection of HR and the true HR signal.

**Supplementary Table 2: R-square score for projection of HR cycles.** Mean values are given with standard deviations shown in brackets.

| **ID** | **daily** | **3d** | **weekly** | **10d** | **fortnightly** | **monthly** | **>60days** |
| --- | --- | --- | --- | --- | --- | --- | --- |
| **1** | 0.95 (0.03) |  |  |  | 0.84 (0.22) | 0.98 (0.03) |  |
| **2** | 0.11 (0.16) |  |  |  |  |  |  |
| **3** | 0.09 (0.14) |  |  | 0.39 (0.29) |  | 0.99 (0.05) |  |
| **4** | 0.8 (0.07) | 0.55 (0.39) | 0.49 (0.42) |  |  | 0.59 (0.43) | 0.80 (0.31) |
| **5** | 0.19 (0.27) |  |  |  |  |  |  |
| **6** |  | 0.84 (0.24) | 0.67 (0.35) | 0.59 (0.34) | 0.86 (0.22) |  |  |
| **7** | 0.54 (0.35) |  | 0.82 (0.01) |  |  | 0.99(<0.01) |  |
| **8** | 0.12 (0.19) |  |  |  |  |  | 0.99(<0.01) |
| **9** | 0.3 (0.34) |  |  |  |  | 0.99(<0.01) | 0.89 (0.22) |
| **10** | 0.24 (0.31) |  |  |  |  | 0.99(<0.01) |  |
| **11** | 0.88 (0.18) | 0.72 (0.14) | 0.97 (0.11) | 0.94 (0.11) |  | 0.98 (0.03) |  |
| **12** |  | 0.29 (0.23) |  |  | 0.99(<0.01) | 0.99(<0.01) |  |
| **13** | 0.11 (0.04) |  |  |  |  |  |  |

**Supplementary Table 3: Mean square error (MSE) for projection of HR cycles.**

| **ID** | **daily** | **3d** | **weekly** | **10d** | **fortnightly** | **monthly** | **>60days** |
| --- | --- | --- | --- | --- | --- | --- | --- |
| **1** | 0.017 |  |  |  | 0.001 | 0.006 |  |
| **2** | 0.100 |  |  |  |  |  |  |
| **3** | 0.110 |  |  | 0.017 |  | <0.001 |  |
| **4** | 0.030 | 0.008 | 0.016 |  |  | 0.009 | 0.064 |
| **5** | 0.100 |  |  |  |  |  |  |
| **6** |  | 0.003 | 0.021 | 0.013 | 0.004 |  |  |
| **7** | 0.050 |  | 0.005 |  |  | <0.001 |  |
| **8** | 0.020 |  |  |  |  |  | <0.001 |
| **9** | 0.100 |  |  |  |  | <0.001 | 0.021 |
| **10** | 0.160 |  |  |  |  | <0.001 |  |
| **11** | 0.020 | 0.008 | 0.001 | 0.004 |  | 0.007 |  |
| **12** |  | 0.013 |  |  | <0.001 | <0.001 |  |
| **13** | 0.080 |  |  |  |  |  |  |

**Supplementary Table 4. Forecast comparison for diary, HR, and combined models.** AUC scores that showed significant improvement compared to chance (p < 0.05 using surrogate method) are shown in bold. The mean AUC values were computed across all participants. The ‘mean of the best forecast’ is the mean of the highest AUC out of diary, HR, or combined forecasts for every participant. Values in brackets indicate data from the evaluation period.

| **ID** | **Hourly AUC** | | | **Daily AUC** | | |
| --- | --- | --- | --- | --- | --- | --- |
|  | **Diary** | **HR** | **Combined** | **Diary** | **HR** | **Combined** |
| **1** | **0.67** (0.69) | 0.56 (0.63) | 0.62 (0.66) | **0.63 (0.65)** | 0.50 (0.39) | 0.53 (0.56) |
| **2** | 0.80 | 0.80 | **0.83** | 0.64 | **0.96** | **0.60** |
| **3** | **0.68** | 0.57 | 0.62 | **0.69** | 0.56 | **0.57** |
| **4** | **0.83 (0.83)** | 0.81 (0.77) | 0.81 (0.81) | **0.69** (0.68) | **0.68** (0.60) | 0.59 (**0.69**) |
| **5** | **0.68** | 0.60 | 0.68 | 0.60 | **0.63** | 0.53 |
| **6** | 0.53 (0.61) | 0.52 (0.59) | 0.57 (0.79) | **0.68** (0.59) | 0.50 (**0.98**) | 0.49 (**0.74**) |
| **7** | **0.76** | 0.55 | 0.75 | **0.66** | 0.50 | **0.62** |
| **8** | 0.61 | 0.61 | 0.58 | 0.49 | **0.68** | 0.42 |
| **9** | 0.70 (0.78) | 0.61 (0.58) | **0.71 (0.78)** | 0.50 (0.48) | 0.49 (0.41) | 0.50 (0.50) |
| **10** | 0.69 (0.78) | **0.82** (0.77) | 0.70 (**0.82**) | 0.55 (0.60) | **0.87** (**0.89**) | 0.51 (**0.61**) |
| **11** | **0.62** | 0.56 | 0.56 | **0.56** | 0.48 | 0.46 |
| **12** | 0.49 (**0.66**) | 0.33 (0.52) | 0.50 (0.50) | 0.56 (**0.71**) | 0.29 (0.55) | 0.53 (0.58) |
| **13** | 0.62 | 0.93 | 0.46 | 0.49 | **1.00** | 0.30 |
| **Mean:** | 0.67 (0.73) | 0.64 (0.64) | 0.65 (0.73) | 0.60 (0.62) | 0.63 (0.64) | 0.51 (0.61) |
| **Mean of Best Forecast:** | 0.71 (0.76) | | | 0.70 (0.74) | | |

**Supplementary Table 5. Performance of time-of-day models.** AUC values for time-of-day models at hourly and daily resolutions, during testing and evaluation periods. The mean AUC values were computed across all participants.

| **ID** | **Hourly AUC** | | **Daily AUC** | |
| --- | --- | --- | --- | --- |
|  | **Testing** | **Evaluation** | **Testing** | **Evaluation** |
| **1** | 0.63 | 0.65 | 0.55 | 0.55 |
| **2** | 0.68 |  | 0.49 |  |
| **3** | 0.55 |  | 0.50 |  |
| **4** | 0.73 | 0.77 | 0.53 | 0.47 |
| **5** | 0.67 |  | 0.42 |  |
| **6** | 0.49 | 0.54 | 0.60 | 0.50 |
| **7** | 0.73 |  | 0.50 |  |
| **8** | 0.70 |  | 0.45 |  |
| **9** | 0.70 | 0.78 | 0.50 | 0.49 |
| **10** | 0.65 | 0.72 | 0.51 | 0.45 |
| **11** | 0.66 |  | 0.53 |  |
| **12** | 0.62 | 0.70 | 0.56 | 0.50 |
| **13** | 0.71 |  | 0.45 |  |
| **Mean AUC** | **0.66** | **0.69** | **0.51** | **0.49** |

**Supplementary Table 6. Brier Skill Scores**

| **ID** | **Hourly BSS** | | | **Daily BSS** | | |
| --- | --- | --- | --- | --- | --- | --- |
|  | **Diary** | **HR** | **Combined** | **Diary** | **HR** | **Combined** |
| **1** | 0.02 (0.03) | 0.00 (0.01) | 0.01 (0.00) | 0.08 (0.19) | 0.01 (-0.03) | 0.04 (0.07) |
| **2** | 0.12 | 0.07 | 0.09 | 0.06 | 0.60 | 0.07 |
| **3** | 0.04 | 0.00 | 0.03 | 0.19 | 0.05 | 0.08 |
| **4** | 0.05 (0.04) | 0.01 (0.01) | 0.01 (0.01) | 0.10 (0.09) | 0.04 (0.00) | 0.05 (0.04) |
| **5** | 0.06 | 0.02 | 0.02 | 0.01 | 0.12 | 0.03 |
| **6** | 0.01 (0.00) | 0.00 (0.00) | 0.00 (0.01) | 0.04 (0.05) | 0.04 (0.02) | -0.08 (0.07) |
| **7** | 0.04 | 0.00 | 0.01 | 0.10 | 0.01 | 0.09 |
| **8** | 0.01 | 0.00 | 0.00 | 0.05 | 0.09 | -0.04 |
| **9** | 0.03 (0.08) | 0.02 (0.02) | 0.03 (0.04) | 0.00 (0.00) | 0.01 (-0.10) | 0.01 (0.00) |
| **10** | 0.03 (0.13) | 0.06 (0.09) | 0.01 (0.05) | 0.03 (0.04) | 0.40 (0.48) | -0.02 (0.02) |
| **11** | 0.04 | 0.01 | 0.01 | 0.02 | 0.07 | -0.07 |
| **12** | 0.00 (0.01) | -0.01 (0.00) | 0.00 (0.00) | 0.03 (0.20) | 0.05 (0.01) | 0.01 (0.01) |
| **13** | 0.00 | 0.04 | -0.01 | 0.07 | 0.74 | -0.42 |
